# Connecting the Dots: Systems Thinking Highlights the Role of the Environment Sector in One Health Operationalization

**DOI:** 10.1002/gch2.202500256

**Published:** 2025-10-24

**Authors:** Alana Hansen, Anne‐Lise Chaber, Simon Reid, Adriana Milazzo

**Affiliations:** ^1^ School of Public Health The University of Adelaide Level 4 Rundle Mall Adelaide South Australia 5000 Australia; ^2^ School of Animal and Veterinary Sciences The University of Adelaide Roseworthy Campus South Australia 5371 Australia; ^3^ School of Public Health The University of Queensland Herston Road Herston Queensland 4006 Australia

**Keywords:** connection, environment sector, one health, operationalization

## Abstract

With One Health (OH) encompassing a holistic view of the interrelated health of humans, animals and environment, strategies based on OH approaches are gaining momentum in the prevention of emerging and re‐emerging infectious diseases. OH is based upon the principles of intersectoral communication, cooperation and collaboration between stakeholders in the human health, animal health and environment sectors. In the face of changes induced by global threats such as climate change, biodiversity loss and urban encroachment into wildlife ecosystems, the environment sector plays an increasingly important role yet is underrepresented in OH operationalization often due to competing stakeholder priorities in animal and human health. Viewing OH through a systems thinking lens identifies the structures (components and relationships) and mental models (assumptions and beliefs) that show the need for stronger connections between OH stakeholders. In this viewpoint, the value of a systems thinking approach in OH that highlights a holistic approach to zoonotic disease prevention is discussed, and the need for interpersonal and intersectoral connections that precede communication and collaboration. In a changing world strengthening connections with the environment sector is now critically important for global pandemic preparedness and in building capacity for early identification of and response to zoonotic health risks.

## Graphical abstract

This perspective highlights the need for a systems thinking approach in One Health to holistically address zoonotic disease prevention, and to strengthen connections with the environment sector for global pandemic preparedness.

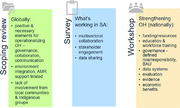

## 1 Introduction

Recent history has shown the importance of acknowledging the interplay between the health of animals and humans, and the need to upscale zoonotic disease prevention and preparedness measures. Emerging infectious diseases in humans, over 75% of which are zoonotic in nature,^[^ ^1^ ^]^ are influenced by population changes in humans and animals, urbanization, international travel and economic conditions.^[^ ^2^ ^]^ One of the most important drivers of zoonotic disease occurrence is anthropogenic land‐use change such as agricultural and urban expansion, which leads to deforestation and habitat degradation that is associated with the spillover of pathogens from animals to humans. For example, the large scale transmission of recent emerging zoonotic diseases such as Zika, Nipah and Ebola viruses have been shown to be directly linked to deforestation and habitat degradation.^[^ ^3^, ^4^ ^]^ In the case of urban sprawl with human encroachment into wildlife habitats there is a heightened risk of pathogen spillover as shared human/animal environments can pose zoonotic disease risks associated with resident species, including mosquitoes,^[^ ^5^ ^]^ nematodes,^[^ ^6^ ^]^ ticks^[^ ^7^, ^8^ ^]^ and bats.^[^ ^9^ ^]^

The recognition of this important interconnectedness of the environment with animal and human health underpins the OH concept which holistically addresses health threats affecting humans, plants, animals and their shared environment.^[^ ^10^ ^]^ With changes occurring in the state of the environment that may have cross‐sectoral health impacts, there is a need for operationalization of OH at global, national, state and local levels.

A OH approach to global health security aims for healthy sustainable socioecological systems by acknowledging sectoral interconnections between humans, animals and the environment.^[^ ^11^ ^]^ Global health security is addressed under the International Health Regulations (IHR) (2005), whereby countries are required to contribute to detect and respond to unusual health events that may be of international concern. In recognition that a OH approach is essential for the implementation of the IHR, the World Health Organization (WHO) established a tripartite between the Food and Agriculture Organization of the United Nations (FAO) and the World Organization for Animal Health (WOAH).^[^ ^12^ ^]^ In 2021, the important association between the environment and health was recognized with the United Nations Environment Program (UNEP) being included in the Tripartite, thus forming the Quadripartite.^[^ ^4^, ^13^ ^]^ To action the OH approach the Quadripartite developed the One Health Joint Plan of Action (OHJPA)^[^ ^10^ ^]^ structured on the Theory of Change (ToC). Using the OH principles of communication, collaboration, coordination and capacity building across the animal, human and environment sectors, the plan comprises three pathways toward operationalization of OH. Pathway 1 concerns governance, policy, legislation, financing and advocacy; Pathway 2 organizational and institutional, development, implementation and sectoral integration; and Pathway 3 data, evidence, information systems and knowledge exchange.^[^ ^14^ ^]^

In this paper we draw on findings of an OHJPA‐based, scoping review, stakeholder survey and workshop in Australia and affirm the importance, yet often underrepresentation, of the environment sector in the OH realm. Further, we posit the need for an extra “C” in the OH principles – *connection* – referring to the interconnection of factors within a system and its subsystems. By mapping and characterizing these interconnections and the stakeholders involved, we argue that the OH system will be more inclusive of the environment sector, and the capacity to detect early spillover events strengthened to aid disease prevention efforts.

## 2 Methods

A suite of studies was conducted to identify global and local examples of OH activities using the OHJPA ToC as an analytical framework.^[^ ^10^ ^]^ These were: 1) a systematic scoping review of OH literature,^[^ ^15^ ^]^ 2) a survey of OH stakeholders and 3) a stakeholder workshop.

### 2.1 Scoping Review

As detailed in Milazzo et al. (2025)^[^ ^15^ ^]^ a search of global literature sourced from PubMed, Embase and Scopus databases yielded 54 studies, with 77 OH programs of which 42 involved the human and animal sectors, while none involved the environment sector.

### 2.2 Stakeholder Survey

In accordance with the OH ToC documentation by the One Health High Level Expert Panel,^[^ ^14^ ^]^ an institutional stakeholder mapping and analysis was undertaken in South Australia (SA) in 2023 to gauge local OH activities and capacity.  An online survey was conducted across government and non‐government organizations with questions underpinned by the OHJPA. Respondents involved in OH practice were selected using cluster sampling and recruited from organizations identified though professional networks. The analysis of closed‐ended questions was undertaken quantitatively, while qualitative responses to open‐ended questions were analyzed using content analysis to identify patterns in the data.

### 2.3 Stakeholder Workshop

In November 2023 in Adelaide, the capital city of SA, a workshop was conducted with stakeholders working in human health, animal health or the environment sector. The objective of the meeting was to elicit their priorities and recommendations on strengthening OH operationalization locally and nationally. The workshop program built upon the survey responses and used the OHJPA ToC conceptual framework.^[^ ^14^ ^]^ Key presentations from expert speakers were followed by individual and group activities and an open discussion session. Proceedings were recorded and the data transcribed for analysis and supplemented with researchers’ notes. Qualitative analysis of the data was undertaken using the Framework Method.^[^ ^16^ ^]^

### 2.4 Ethical Approval and Consent to Participate

The study was approved by the SA Department for Health and Wellbeing Human Research Ethics Committee (2023/HRE00174) and endorsed by the University of Adelaide Ethics Committee. Written consent was obtained from participants prior to commencement of the workshop.

## 3 Results and Discussion

### 3.1 Scoping Review

The reviewed studies mostly incorporated the ToC Pathway 2 (organizational and institutional development, implementation and sectoral integration), and Pathway 1 (governance, policy, legislation, financing, and advocacy), followed by with fewer focussing on Pathway 3 (data, evidence, information systems, and knowledge exchange). Importantly, we identified a minimal engagement of the environment sector in OH implementation with only 20.8% of the programs reviewed integrating environmental concerns such as climate change and impacts on ecosystems. The study also noted that greater incorporation of the environmental considerations in OH strategies may be beneficial in the fight against antimicrobial resistance.^[^ ^15^ ^]^

### 3.2 Stakeholder Survey

Of the 45 survey respondents from 12 organizations, those from the environmental sciences sector comprised just under 50%, perhaps due to the government‐focused recruitment process. The respondents identified that poor intersectoral communication bsetween organizations was a key barrier to OH collaborations; and more than two thirds reported no engagement with OH networks despite most undertaking OH activities. The findings also indicated local level involvement and stewardship would be beneficial with inclusion of local government in OH operations.

### 3.3 Stakeholder Workshop

For Pathway 1 (governance, policy, legislation, financing, and advocacy) attendees discussed topics including the recreation, health and monetary value of ecosystems and their protection. For Pathway 2, (organizational and institutional development, implementation, and sectoral integration) participants voiced the need for community engagement and good OH governance that includes animal health and health of ecosystems. An attendee from the animal health sector highlighted the need for governance with equity in representation across sectors: *“I think it really needs a strategic plan, and a governance model, an implementation plan … and it needs to be done equitably between all … partners.”* For Pathway 3 (data, evidence, information systems, and knowledge exchange) discussions focussed on knowledge translation, strengthening of collaboration between actors and intersectoral communication regarding accessibility of disease surveillance and outbreak data. Overall, there was triangulation in the findings from these studies, which point to the underrepresentation of the environment sector in OH operationalization and the need to re‐think intersectoral connections for optimum benefits at the human health‐animal‐environment interface.

### 3.4 Connecting the Dots Using Systems Thinking

A consistent theme of our studies was that without coordination, collaboration, communication and an equitable governance model, the system fails. The important role each sector plays within the OH triad argues for better connectedness within a holistic system. This prompts the need to approach OH operationalization through a systems thinking lens.

Systems thinking views a system holistically by examining its structure and interconnected components, their patterns of behavior and context, and the mental models that underlie the structure.^[^ ^17^, ^18^ ^]^ Systems thinking is an ideal way to examine complex issues such as the dynamics of infectious disease transmission and intervention measures,^[^ ^17^, ^19^ ^]^ antimicrobial resistance^[^ ^20^ ^]^ and OH.^[^ ^18^, ^21^ ^]^ Xie et al (2017)^[^ ^18^ ^]^ listed steps in applying a systems thinking approach to understand a complex problem, including: problem identification; behavior analysis to identify components within the system and their influence on each other; model hypothesis using causal loop diagrams (CLDs); model evaluation ensuring correct presentation of relationships; and concluding with a discussion of the system.^[^ ^18^ ^]^ The systems thinking approach and development of CLDs is enhanced through group model‐building processes that are informed by stakeholder input.^[^ ^21^ ^]^ This enables a qualitative form of system mapping to show interconnections and feedback relationships (positive and negative) between system factors.^[^ ^21^ ^]^ This approach was recently used to develop a simulation model to develop OH interventions for the neglected zoonotic disease brucellosis.^[^ ^19^ ^]^

### 3.5 Connections Amongst Stakeholders

In terms of OH, the OHJPA principles foster the strengthening of “collaboration, communication, capacity building and coordination” equally across the sectors involved in health at the human‐animal‐environment interface.^[^ ^10^ ^]^ However, we contend that there is a missing “C” – *connection* – that is required between the sectors, particularly among the stakeholders. Connection differs slightly from coordination and collaboration, which both require high levels of interdependency. Keast and Mandell (2014)^[^ ^22^ ^]^ argue that coordination brings together interdependent parts, for example, separate organizations contributing to an agreed outcome. Collaboration requires reciprocal interdependency, with reliance and dependence on another organization or stakeholder to address a common problem.^[^ ^22^ ^]^ Connection however, differs from the existing four Cs in that it can refer to relationships built on shared interests or common concerns. These connections may be social relationships that can help progress initiatives, bring new insights to the table, and help influence decision making.^[^ ^23^ ^]^ OH networks are an ideal example of informal connections between stakeholders. The Regional One Health Partnership, based in the Australian state of New South Wales, is one such example with members who have shared interests in animal, human and environment health and zoonotic disease prevention.^[^ ^24^ ^]^ There can be numerous connections within a network as demonstrated by a stakeholder and social network analysis of a OH network in the Netherlands which found 153 connections between 47 stakeholders across all domains and governance levels.^[^ ^25^ ^]^

A case study of the benefits of stakeholder connections in OH operationalization was highlighted at the stakeholder workshop in SA, mentioned previously. Attendees from a OH working group discussed the response to a local Japanese encephalitis virus outbreak,^[^ ^26^ ^]^ its implications for animal (pigs) and human health, and the importance of the mosquito surveillance data in the response. The members reported that government departments cooperated well, and the necessary data were promptly acquired enabling timely scaling up of fieldwork and surveillance. This was made possible by the existing relationships between stakeholders as noted by an attendee from the human health sector: “*Because … members in South Australia were very familiar with membership from all those government departments we cooperated reasonably well, … the data that we managed to acquire from … for example which was crucial…[and] collecting specimens and expanding surveillance, … we managed to achieve that pretty quickly*.”

Stakeholder connections offer several benefits that complement coordination and collaboration. These benefits encompass inclusion of diverse perspectives, flexibility and agility, broader resource access, and a sense of community and support. Connections via increased informal communication between stakeholders can be of benefit to interorganizational relationships with the level of formality or informality varying according to the linkage relationships.^[^ ^27^ ^]^ Studies have shown that enhanced connections between agencies can build trust and reciprocity whether in governments or at the community level, leading to improved outcomes.^[^ ^22^ ^]^ Connections create networks that include a wider variety of stakeholders, bringing in different viewpoints and insights that may not be present in more formal coordinated efforts. Established trusting connections through social networks allow informal information sharing, which may increase the speed of idea exchange, innovation and communication of issues or updates. This, in turn, enables more flexible relationships where stakeholders can adapt quickly to changing situations without the constraints of formal agreements typical in coordination and collaboration processes. Furthermore, access to scarce resources may occur via the leverage inherent in a social network of connections. Finally, strong connections build a sense of community among stakeholders, leading to mutual support and potentially easing collaboration and coordination when needed.

Identification and development of personal connections within and between stakeholders in different sectors is an essential component of the operationalization of OH to ensure the system addresses intersectoral health issues. Stakeholder identification, mapping and analysis^[^ ^14^, ^19^ ^]^ is a particularly important tool in a systems thinking approach for identifying disease‐specific drivers, planning effective interventions or identifying early signs of spillover events. For zoonotic disease investigations, stable connections between high‐level government departments are key in OH governance. However, stakeholders at the grassroots level within the sectors will likely vary considerably at the non‐government and community level according to the zoonotic disease under consideration and its pathogen reservoirs.

### 3.6 The Need for Better Connections with the Environment Sector

Stakeholder identification must be approached broadly and inclusively in complex systems as the main sectors are not always mutually exclusive or easily identified. This is particularly important in the environment sector because of varied interpretations of the terms “environment” and “environmental health”. Often used in relation to the health of the environment, “environmental health” specifically refers to a public health function to assess and control environmental factors that can potentially affect human health. At the local government level, environmental health officers are OH practitioners whose multisectoral roles cover animal, human and environment sectors, for example: environmental protection, food safety, climate change, animal management, infection control, monitoring and surveillance and environmental sampling.^[^ ^28^, ^29^ ^]^

Depending on the nature of the zoonotic disease, other stakeholders in the “environment sector” may include: Indigenous peoples, citizen scientists including community members in country and urban areas, academics and researchers, wildlife experts, park rangers, ecologists, urban planners, climate scientists, agriculturalists, farmers, entomologists, environmental economists, environmental scientists, conservation groups, and the list goes on. In the case of emerging infectious diseases, social environments also need to be considered as part of the systems, with acknowledgement of the connections with socio‐economic and socio‐political drivers and impacts of the diseases.^[^ ^30^ ^]^

With such a broad scope in interpretation of “environment”, identifying and integrating relevant stakeholders from this sector in OH operationalization can be challenging. This may contribute to the identified underrepresentation of stakeholders from the environment sector in OH networks.^[^ ^24^, ^25^ ^]^ An identified barrier to integrating these actors is that those working in the environmental sciences can “struggle to connect the relevance of their work with the One Health paradigm”.^[^ ^31^ ^]^ If the main focus of a project is communicable disease burdens in animals and humans, environment sector actors may be influenced by siloed governance mechanisms. Legislative frameworks for communicable disease control in Australia at different levels of government (Commonwealth, state and local)^[^ ^32^ ^]^ necessarily centre on the public health aspects which may be a disincentive for the inclusion of environment sector actors. To address the underrepresentation of those from the environment domain in OH, collaborations and connections among existing stakeholders in networks and organizations could be utilized to promote the importance of sectoral equality in OH operations.^[^ ^25^ ^]^

### 3.7 How Intersectoral Connections can Aid in Disease Prevention

In OH, transdisciplinary connections are increasingly necessary for the control and prevention of emerging infectious diseases^[^ ^30^ ^]^ and identifying early signs of spillover. Spillover of zoonotic pathogens from animals to humans is the most common cause of emerging or re‐emerging infectious diseases,^[^ ^1^ ^]^ and may become more common due to changing environmental and social conditions. However, there is currently no reliable means of detecting the early signals of spillover as concerns of a new zoonotic outbreak are usually raised when the first human cases appear. Systems thinking, incorporating stakeholder mapping and the development of disease‐specific CLDs could aid in the early detection of the drivers and signals of potential zoonotic spillover events.

A bottom‐up approach to surveillance encompassing all relevant stakeholders can be useful in detecting anomalies in environment or animal health at the source of disease emergence^[^ ^33^ ^]^ before human infection occurs. These anomalies may include, for example, observations of sick birds including domestic poultry or wildlife, the laboratory detection of arboviruses in mosquitoes or sentinel chickens, diagnoses of zoonoses in farmed animals,^[^ ^33^ ^]^ or virus detection in wastewater samples.^[^ ^34^ ^]^

There are many other examples where the integration of environmental data can boost OH intelligence to aid in disease prediction, prevention and preparedness.^[^ ^35^ ^]^ This is particularly relevant with respect to climate change as many studies have shown associations between climate variables and mosquito‐borne diseases, with higher temperatures and changed rainfall patterns affecting the geographic range of the vectors, mosquito breeding rates, adult lifespan, and pathogen replication rates.^[^ ^36^ ^]^ For instance, environmental data (climate data and mosquito surveillance data) have been combined with disease notification data to increase the sensitivity for the prediction of epidemics of climate sensitive arboviral diseases such as Ross River virus.^[^ ^11^, ^37^, ^38^ ^]^

Increases in extreme climate events are predicted to affect zoonotic disease risk via changes in animal and human migration patterns^[^ ^3^, ^36^ ^]^ that may lead to novel interactions between species as has occurred with Hendra virus.^[^ ^3^ ^]^ Flying foxes, the reservoir of Hendra virus in Australia, have migrated southward in recent years owing to changes in climatic conditions and land‐use change, resulting in virus spillover to horse populations and humans.^[^ ^3^ ^]^ Other zoonotic diseases including those transmitted to humans via rodents, ticks, flies and water snails^[^ ^36^, ^39^ ^]^ are also affected by climate change and fall within the OH paradigm, prompting the need for field and climate data to supplement standard surveillance methods in monitoring disease risks at the environment‐human‐animal interface.

Although combining and synthesizing data from heterogeneous sources can be challenging, purpose‐built surveillance systems integrating for example environmental (hazard exposures, climate and air pollution data), animal (livestock, wildlife, domestic, sentinel animals) and human (qualitative, laboratory, clinical) data have been designed.^[^ ^40^ ^]^ However, there are often confidentiality, legal, ethical and technical barriers and challenges with harmonizing data from multiple domains and in different formats.^[^ ^11^, ^40^ ^]^ Artificial intelligence (AI) has been used in infectious disease surveillance, and forecasting of new disease outbreaks^[^ ^41^ ^]^ and it is expected that this technology will soon be used to solve some of the technical issues of data harmonization making way for a truly integrated OH surveillance system able to detect early environmental signals that may predict and perhaps avert emerging and re‐emerging infectious disease outbreaks.

Sentinel surveillance can indicate a possible emerging threat to health and if signals are detected the first responses should involve stakeholder connections to enable collaboration in the exploration of the issues and determine the likelihood of false positive signals. In the event of a human outbreak the stakeholder realm of connections would likely escalate to include general practitioners, hospital staff, laboratory personnel, epidemiologists and health departments. The results of diagnostic tests and genetic sequencing would need to be rapidly communicated via data surveillance networks to identify the disease source, transmission patterns and vulnerable populations.^[^ ^42^ ^]^ This information and data sharing would need to be part of the necessary intersectoral connections preceding communication, coordination, and collaboration in the governance of OH responses. Therefore, it is important to ensure that mechanisms are in place to reduce health risks, and predict, detect and respond quickly should they occur.^[^ ^43^ ^]^ Having existing disease‐specific stakeholder maps to visualize the system dynamics, interconnectedness and contributing factors to zoonotic spillover events^[^ ^21^ ^]^ would make these mechanisms more streamlined and build capability to prevent escalation of disease transmission.

## 4 Conclusion

In this paper we have provided convincing evidence of the need to connect the dots in OH frameworks to minimize siloed thinking around the three individual domains (animal health, human health, environment health). When OH is viewed through a system thinking lens the interconnections within and between stakeholders and subsystems become evident.

Although the guiding principles of OHJPA 2022‐26 include inclusivity and equity of all stakeholders; and communication, collaboration and coordination across sectors^[^ ^10^ ^]^ there is no specific emphasis on “connection”, which we suggest could be considered in the next iteration of the OHJPA. Moreover, based on the findings of our own studies and those reported in the wider literature, we have shown there needs to be greater connections with actors in the environment sector due to the centrality of the environment in the OH paradigm.^[^ ^11^ ^]^ These connections should be actioned across all three tiers of government as well as at the community level, to engender greater sectoral equity in OH participation and governance.

Furthermore, greater involvement of the environment sector in OH could facilitate better integration of environmental data into surveillance systems to increase the efficacy of OH preparedness efforts, particularly in light of major environmental threats such as climate change, habitat destruction, and biodiversity loss.^[^ ^15^ ^]^

In summary, we have provided a novel contribution to the OH perspective and identified a gap that if filled may strengthen operationalization efforts. We therefore recommend that:

1. Building on the existing OH principles of communication, coordination and collaboration there be greater emphasis on connections and interconnections within the OH paradigm. This includes connections between: (a) sectors, (b) stakeholders, and (c) causal factors in the system dynamics of disease transmission.
2. Efforts be made to increase the participation of stakeholders in the environment sector. This should be undertaken using a broad and flexible definition of “environment” that may include aspects of the natural, built, social, economic and socioecological environments that can influence the health of animals, humans and ecosystems.
3. Environmental surveillance data be included into national OH surveillance platforms to boost OH intelligence and aid in identifying potential environmental triggers for pathogen spillover.

## Conflict of Interest

The authors declare no conflict of interest.
